# Gender transformative approaches in mHealth for maternal healthcare in sub-Saharan Africa: a systematic review

**DOI:** 10.3389/fdgth.2023.1263488

**Published:** 2023-11-07

**Authors:** Ogochukwu Udenigwe, Olumuyiwa Omonaiye, Sanni Yaya

**Affiliations:** ^1^ School of International Development and Global Studies, University of Ottawa, Ottawa, ON, Canada; ^2^ Centre for Quality and Patient Safety Research, Institute for Health Transformation, Deakin University, Burwood, VIC, Australia; ^3^ Centre for Quality and Patient Safety Research—Eastern Health Partnership, Eastern Health, Box Hill, VIC, Australia; ^4^ The George Institute for Global Health, Imperial College London, London, United Kingdom

**Keywords:** digital health, mHealth, maternal health, gender integration, gender transformative, sub-Saharan Africa

## Abstract

**Background:**

This review focuses on studies about digital health interventions in sub-Saharan Africa. Digital health interventions in sub-Saharan Africa are increasingly adopting gender-transformative approaches to address factors that derail women's access to maternal healthcare services. However, there remains a paucity of synthesized evidence on gender-transformative digital health programs for maternal healthcare and the corresponding research, program and policy implications. Therefore, this systematic review aims to synthesize evidence of approaches to transformative gender integration in digital health programs (specifically mHealth) for maternal health in sub-Saharan Africa.

**Method:**

The following key terms “mobile health”, “gender”, “maternal health”, “sub-Saharan Africa” were used to conduct electronic searches in the following databases: PsycInfo, EMBASE, Medline (OVID), CINAHL, and Global Health databases. The method and results are reported as consistent with PRISMA (Preferred Reporting Items for Systematic Reviews and Meta-Analyses). Data synthesis followed a convergent approach for mixed-method systematic review recommended by the JBI (Joanna Briggs Institute).

**Results:**

Of the 394 studies retrieved from the databases, 11 were included in the review. Out of these, six studies were qualitative in nature, three were randomized control trials, and two were mixed-method studies. Findings show that gender transformative programs addressed one or more of the following categories: (1) gender norms/roles/relations, (2) women's specific needs, (3) causes of gender-based health inequities, (4) ways to transform harmful gender norms, (5) promoting gender equality, (6) progressive changes in power relationships between women and men. The most common mHealth delivery system was text messages via short message service on mobile phones. The majority of mHealth programs for maternal healthcare were focused on reducing unintended pregnancies through the promotion of contraceptive use. The most employed gender transformative approach was a focus on women's specific needs.

**Conclusion:**

Findings from gender transformative mHealth programs indicate positive results overall. Those reporting negative results indicated the need for a more explicit focus on gender in mHealth programs. Highlighting gender transformative approaches adds to discussions on how best to promote mHealth for maternal health through a gender transformative lens and provides evidence relevant to policy and research.

**Systematic review registration:**

PROSPERO CRD42023346631.

## Introduction

At the 71st World Health Assembly in 2018, resolutions on digital health underscored the need for digital health to not only enhance existing health service delivery models but to also contribute towards achieving health equity including gender equality (1). Precedents on gender integration in women's health were set in the 1990s and addressed the broad category of health issues that are unique to women such as maternal health and health issues that may manifest differently in women than men such as heart diseases. Significant global gatherings such as the International Conference on Population and Development and the World Conference on Women in Beijing recognized gender inequality as a critical factor influencing health, particularly for women who face disproportionate disadvantages in health outcomes (2, 3). Women face unequal access to healthcare resources and bear the burden of gender stereotypes that are perpetuated through health policies and programs, this had resulted in inadequate or inappropriate services for women (2–4). Targeting gender attitudes and norms is an important part of the broader strategy to achieve the sustainable development goals, but explicit attention to gender is often missing in health programming.

**Figure 1:**
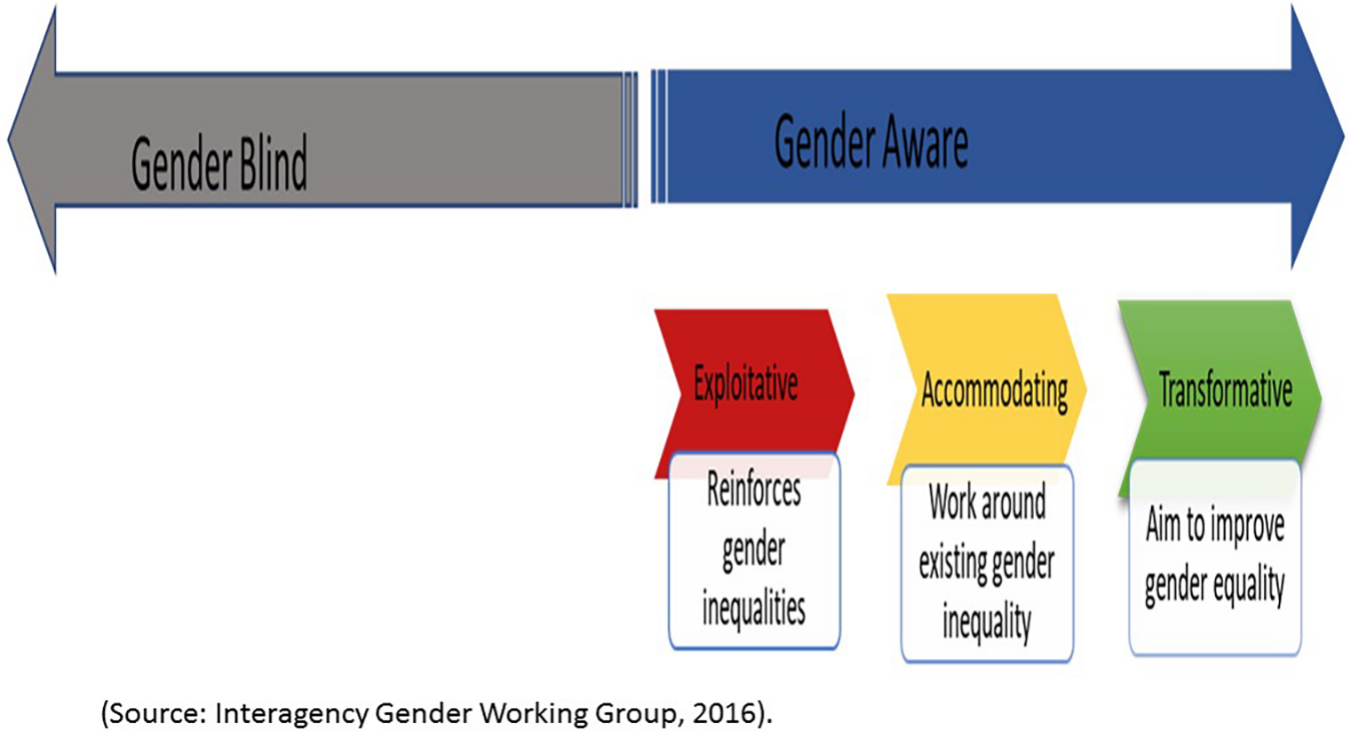
Gender integration continuum.

Aligned with sustainable development goal (SDG) 5 which aims to *Enhance the use of enabling technology, in particular information and communications technology, to promote the empowerment of women*, digital health is showing the potential to drive gender equality by reducing unequal access to and use of healthcare services (5). The field of digital health focuses on the use of information and communication technologies systems or channels, software, and data to improve health and wellness (6). While the healthcare transformations brought about by digital health are fundamentally technological, digital health also transforms the social, economic, and political context within which they occur (7, 8). Therefore, digital health programs must foreground the voices and realities of users, especially marginalized populations in their program design and delivery. Digital health has been incorporated across the pregnancy care continuum in efforts to address social determinants of health, improve the quality of care and ensure positive maternal health outcomes (9–11). However, to achieve meaningful impact, gender and digital inclusion must remain a priority in developing, implementing and evaluating digital health programs. Women, who are often the target groups for maternal health programs, are not homogenous. Their social, cultural and structural context will differ based on the relationships that govern their everyday lives (7). Barriers brought about by gender dynamics have demand and supply-side implications for women's participation in digital health for maternal healthcare. On the demand side, for example, the gender divide in mobile phone ownership or unfavourable community and cultural preferences, attitude, and norms around women's participation in digital health can impede women's participation in digital health and even exacerbate existing inequalities in access to digital health services (12, 13). On the supply side, breaches of confidentiality of women's health data on digital health programs or culturally insensitive digital health programs are detrimental to women's participation in digital health (14, 15). These implications demonstrate the importance of sex and gender considerations in digital health programming especially for maternal health. Considering gender in and of itself is not sufficient because some pathways to gender consideration in health can exploit or accommodate harmful gender norms rather than transform them. Figure 1 depicts different types of gender inclusion strategies and serves as a guide for discussions on the implications of these strategies on gender equity outcomes.

Gender-blind health programs have no gender considerations, they ignore gender norms and relations and consequently risk reinforcing gender-based discrimination, biases, and stereotypes. Gender-aware programs, on the other hand, acknowledge gender norms and adopt an approach along a continuum as follows: First, gender exploitative approaches intentionally or unintentionally take advantage of gender inequalities to advance program outcomes thereby exacerbating gender inequalities; Second, gender-accommodating programs acknowledge but circumvent gender inequalities to achieve program outcomes; Third, gender transformative approaches in health programming aim to change gender power dynamics and/or reduce gender gaps in access to resources to achieve equitable gender norms and dynamics (16–18). Such gender-informed implications are integral to understanding how to approach health intervention efforts for maternal health.

The need for gender transformative approaches in health programming is increasingly highlighted in global health research, especially as it pertains to maternal healthcare (4, 19). This need recognizes gender as a key determinant of maternal health and acknowledges that women and girls are disproportionately disadvantaged in health outcomes. Gender transformative approaches in non-digital health programs have been shown to be effective in improving maternal health. For example, integrating gender-specific differences in health promotion measures across sub-Saharan Africa led to shifts in gendered attitudes and behaviours which in turn improved maternal health outcomes (4). In Rwanda, an intervention that tackled inequitable power dynamics within heterosexual relationships saw increased modern contraceptive use among women and increased men's engagement in pregnancy care (20). In Uganda, a gender transformative approach to prevent violence against women and prevent HIV risk saw shifts in deeply entrenched attitudes on inter-partner violence among men and women (21).

Digital health interventions in sub-Saharan Africa are also adopting gender transformative approaches to address factors that derail women's access to maternal healthcare services. For instance, in Kenya, a mHealth program identified a digital divide within their target population and implemented strategies to improve women's digital access to quality maternal health services (22). Their strategies included the provision of inexpensive mobile phones, digital literacy for women, and working with men and the community to address negative social norms that restrict women's access to digital technology. In Nigeria, studies showed that addressing women's specific needs such as increased access to required technology improves women's participation in digital health programs and maternal health outcomes (23, 24).

Identifying such gender transformative approaches will inform policy and enhance best practices for gender integration in digital health. However, there remains a paucity of synthesized evidence on gender transformative considerations being made in digital health programs for maternal healthcare in sub-Saharan African contexts and the corresponding research, program and policy implications. Therefore, this systematic review aims to synthesize evidence of approaches to transformative gender integration that address gender inequality in mHealth for maternal health in sub-Saharan Africa. Addressing gender inequality in health programming is conceptualized as a gender-transformative approach. This review adopts the definition offered by the World Health Organization (WHO) and interprets a gender-transformative digital health program as one that “addresses the causes of gender-based health inequities through approaches that challenge and redress harmful and unequal gender norms, roles, and power relations that privilege men over women (18) (p. 136)”.

This systematic review will address the following questions:

1. How are mHealth interventions for maternal health in sub-Saharan Africa adopting gender transformative approaches?
2. To what extent are gender-transformative interventions positively impacting maternal health outcomes?

## Method

This review has been registered on PROSPERO with the registration ID CRD42023346631. A review protocol for this review was not prepared. The reporting of this review follows the guidelines outlined in the Preferred Reporting Items for Systematic Reviews and Meta-Analyses (PRISMA 2020) statements (25) (Supplementary File S1). We took a systematic approach to identify peer-reviewed articles where a mHealth intervention for maternal health was designed and implemented in a sub-Saharan African country. Studies were identified by searching for articles published between 2010 and 2021. Date limits were set in congruence with the widespread adoption of digital health foundations (such as programs, strategies and policies) across sub-Saharan African countries (26).

### Eligibility criteria

We sought to identify studies reporting primary evidence regarding digital health for maternal healthcare, thus, we included research that examined the implementation, distribution and evaluation of digital health programs for maternal healthcare. We included peer-reviewed journal articles without restrictions on the study type therefore quantitative, qualitative, and mixed-method studies were included. Maternal health refers to the health of women during pregnancy, childbirth and the postnatal period, therefore our targets were programs or interventions aiming to improve the uptake of services during pregnancy, childbirth, and post-partum follow-up which also reported gender transformative considerations such as consideration for gender roles. We also sought out studies that were conducted in a sub-Saharan country and limited the language to English due to the authors' language proficiencies.

We focused on studies that targeted women and/or men as end users, therefore studies targeting healthcare workers were excluded. We also excluded studies whereby mobile devices were only used for data collection purposes because we wanted the focus to be on devices used for intervention purposes. We did not include studies that were discussing the literature for the purpose of theory building or critique. The inclusion and exclusion table is available as a supplementary document (Supplementary File S3).

### Search strategy

Five databases were searched from 2010 to September 2021. The databases are PsycInfo, EMBASE, Medline (OVID), CINAHL, and Global Health. We conducted test searches between September 2020-December 2020 and iteratively adjusted and refined the search strategy. We conducted initial searches in February 2021 and updated them in September 2021. Examples of search terms included “mobile health”, “gender”, “maternal health”, and “sub-Saharan Africa”. We also used synonyms, truncations, and wildcards. The electronic search strategy for the Medline (OVID) database is available as a supplementary document (Supplementary File S2).

### Data extraction and appraisal

Studies included in this review were independently screened by two reviewers (OU and OO). The software Covidence (27) was used to organize and screen each study's title and abstract. The two reviewers subsequently conducted full text reviews of the selected studies. They assessed and resolved conflicts jointly or in consultation with the third author (SY). The two reviewers extracted the relevant data using a data extraction form developed purposely and piloted prior to review. Relevant data from quantitative and qualitative studies were collated and reported on the form. Relevant information included study design, type of mHealth intervention, study aim, intervention outcomes, findings, and limitations. We illustrated gender transformative approaches by adapting the definition of gender transformative programming into six categories as provided by WHO (18), they included ways in which programs; (1) consider gender norms/roles/relations, (2) consider women's specific needs, (3) address the causes of gender-based health inequities, (4) include ways to transform harmful gender norms, (5) seek to promote gender equality, (6) have strategies to foster progressive changes in power relationships between women and men. We were also open to including relevant data that did not fall within the WHO's definition, but we were able to align the extracted information with the predefined categories. See Table 1 for gender consideration categories.

**Table 1:** Gender transformative considerations.

Author, Year	Considers gender norms/roles/relations	Considers women's specific needs	Addresses the causes of gender-based health inequities	Includes ways to transform harmful gender norms	Seeks to promote gender equality	Strategies to foster progressive changes in power relationships between women and men
Ampt et al., (28)		The mHealth intervention was co-designed and tested with self-identified female sex workers from the target population.		The mHealth intervention acknowledges gender-based violence was likely as a result of participating in the mHealth intervention. To safe guard women, intervention ensured counseling, urgent medical treatment and protection by the community		
Dev et al., (29)		The authors identified women's limited knowledge on family planning (FP). The authors developed a FP decision aid designed to help prepare postpartum women to make personalized de- liberated contraceptive choices.			The FP program was designed to narrow the knowledge gap on family planning between men and women.	
Flax et al., (30)		The authors conducted a study apriori and identified that only 11% of women had phones. The mHealth program was designed to address cell phone gaps and enhance access to mHealth interventions by providing a group cell phone messaging intervention to promote optimal breastfeeding practices. Therefore women were able to participate even without individual phone ownership.			The mHealth program was offered as a multi- component program to improve women's financial independence through a microcredit program	
Harrington et al., (31, 32)			SMS messaging was designed to challenge personal subjective and social norms about postpartum pregnancy risk and contraceptive use.	The study was guided by women's emphasis on the need to educate men about FP in order to improve women's FP access.		The mHealth program took an innovative strategy to promote couple FP education and subsequently support couple decision-making through SMS messaging. Men provided feedback on the need to think beyond the woman-spouse dyad and include community-level engagement in FP.
Isler et al., (33)	The study considered gender norms such as the division of labour along gender lines resulting in domestic responsibilities for women. The intervention took tablets to women's door steps to show them educational videos on maternal and child nutrition.	In evaluating the mHealth program, The authors planned data collection activities around cooking hours to allow for mothers to fulfil their household duties. Data collection took place in nearby health centres or participants’ homes to avoid mobility issues. In recognition of participants’ childcare responsibilities, childcare provisions were made for women during focus group discussions as needed.	mHealth showed that it is essential to involve male partners in mHealth maternal nutrition interventions as a means of facilitating the implementation of nutritional advice and fostering constructive couple communication.			
Lund et al., (34)		The intervention design included women regardless of mobile phone and literacy status. This approach was chosen because the voucher component allowed all women, regardless of mobile phone status, access to emergency obstetric care, which the authors felt unethical to limit.				
Onono et al., (35)		mHealth Intervention provided decision-making support because the authors identified decision-making for pregnancy and childbirth service care-seeking as a complex behavior influenced by individual, family, societal, access, and health system factors.				
Parkes-Ratanshi et al., (36)			The study identified untreated men (partners to pregnant women) as primary drivers of Syphylis in pregnant women. This study aimed to increase the testing and treatment of pregnant women's male partners to reduce pregnant women's risk of syphilis.			
Schwartz et al., (37)		The content of the intervention ensured confidentiality by not disclosing women's HIV status. Messages were focused on counselling and support. Women that did not have partner support disclosed that the intervention was particularly important for them and met their needs.				
Skinner et al., (38)		There was no cost for women to participate in the mHealth program. If a woman did not own a phone, messages were sent to another phone where she could read them.		Women indicated that the messages provided a base for discussion. The sharing of certain messages, such as around domestic violence, left the women feeling supported. Messages were shared with expectant fathers, close friends and colleagues.		
Trafford et al., (39)						Women participants attributed low levels of breastfeeding to social norms. The male gaze which indicated men's disapproval of women breastfeeding in public was cited as a reason for not breastfeeding. The messages from the mHealth program enabled women to resist pressure. Women also shared the messages with male relatives to prove the importance of breastfeeding.

The reviewers appraised the quality of the manuscripts using the Mixed Methods Appraisal Tool (MMAT) (40). This tool enabled the appraisal of different classes of research including quantitative research, qualitative research, and mixed-method studies. In assessing the methodological quality of data, the tool examines the appropriateness of data collection methods, the concurrency between the study aims and data collection methods, the sample choice and the interpretation of results. We did not exclude articles based on quality scores alone because critically appraising mixed research studies remains controversial given the complexities involved (41–43). We, however, adhered to recommendations by Hong et al. (40), whereby studies not meeting the screen criteria (Supplementary File S1 and S2) were not considered appropriate for appraisal. All 11 studies passed the screening. For each study design, scores were allotted percentages based on a methodological scoring system where possible items are divided by affirmative items (44, 45). Quality scores of each study were classified as weak (<50%), moderate (50%–80%) and strong (>80%). Screening questions were not allotted percentages. While Hong et al., (40) discourage an overall calculation of scores using the MMAT, we sought to provide a representation of ratings to inform the quality of studies included in this review.

Overall, the quality of the studies ranged from 0% (none of the criteria were met) to 100% (all the criteria were met). The qualitative studies were generally moderate to strong. One of the mixed-method studies was classified as weak for not meeting any of the criteria (37). The randomized control trial studies generally showed the risk of performance bias, this means that outcome assessors may have been aware of the applied intervention which could unconsciously or intentionally alter their assessment (46). Quality appraisals are detailed in Table 2.

**Table 2:** (A) quality appraisal of qualitative studies. (B) Quality appraisal of quantitative studies. (C) Quality appraisal of mixed method studies.

Screen			Qualitative studies					Quality score
(A) Quality appraisal of qualitative studies								
	S1. Are there clear research questions?	S2. Do the collected data allow to address the research questions?	1.1. Is the qualitative approach appropriate to answer the research question?	1.2. Are the qualitative data collection methods adequate to address the research question?	1.3. Are the findings adequately derived from the data?	1.4. Is the interpretation of results sufficiently substantiated by data?	1.5. Is there coherence between qualitative data sources, collection, analysis and interpretation?	
Dev et al., (29)	✓	✓	✓	✓	✓	✓	✓	100%
Harrington et al., (31, 32)	✓	✓	✓	✓	✓	✗	✓	80%
Isler et al., (33)	✓	✓	✓	✓	✗	✗	✓	60%
Onono et al., (35)	✓	✓	✓	✓	✓	✓	✓	100%
Skinner et al., (38)	✓	✓	✓	✓	✓	✓	✓	100%
Trafford et al., (39)	✓	✓	✓	✓	✓	✓	✓	100%
Screen			Quantitative randomized control trials					Quality score
(B) Quality appraisal of quantitative studies								
	S1. Are there clear research questions?	S2. Do the collected data allow to address the research questions?	2.1. Is randomization appropriately performed?	2.2. Are the groups comparable at baseline?	2.3. Are there complete outcome data?	2.4. Are outcome assessors blinded to the intervention provided?	2.5 Did the participants adhere to the assigned intervention?	
Ampt et al., (28)	✓	✓	✓	✓	✓	✗	✓	100%
Lund et al., (34)	✓	✓	✓	✓	✓	✗	✗	60%
Parkes-Ratanshi et al., (36)	✓	✓	✓	✗	✓	✗	✗	40%
Screen			Mixed method studies					Quality score
(C) Quality appraisal of mixed method studies								
	S1. Are there clear research questions?	S2. Do the collected data allow to address the research questions?	5.1. Is there an adequate rationale for using a mixed methods design to address the research question?	5.2. Are the different components of the study effectively integrated to answer the research question?	5.3. Are the outputs of the integration of qualitative and quantitative components adequately interpreted?	5.4. Are divergences and inconsistencies between quantitative and qualitative results adequately addressed?	5.5. Do the different components of the study adhere to the quality criteria of each tradition of the methods involved	
Flax et al., (30)	✓	✓	✓	✗	✓	✗	✓	60%
Schwartz et al., (37)	✓	✓	✗	✗	✗	✗	✗	0%

## Data analysis and synthesis

Data synthesis followed a convergent approach for mixed-method systematic review recommended by JBI (47). The review questions can be answered by both quantitative and qualitative studies therefore data synthesis involved data transformation by way of qualitizing. Qualitizing involves extracting data from quantitative studies and converting them to textual descriptions to allow integration with qualitative studies (47). Data were extracted using a data extraction form that collects information on the study design, type of mHealth intervention, study aims, intervention outcomes, findings, and limitations. The synthesized data are arranged in tabular forms to allow for comparison of the different approaches to gender transformative integration. The authors classified studies into subgroups according to the gender transformative categories identified within the studies. There is a global policy interest in addressing gender inequality in health programming (16). Highlighting the different approaches separately is important because it will add to the discussions on how best to promote mHealth for maternal health through a gender transformative lens and will provide evidence relevant to policy and research.

## Results

### Study characteristics

**Figure 2:**
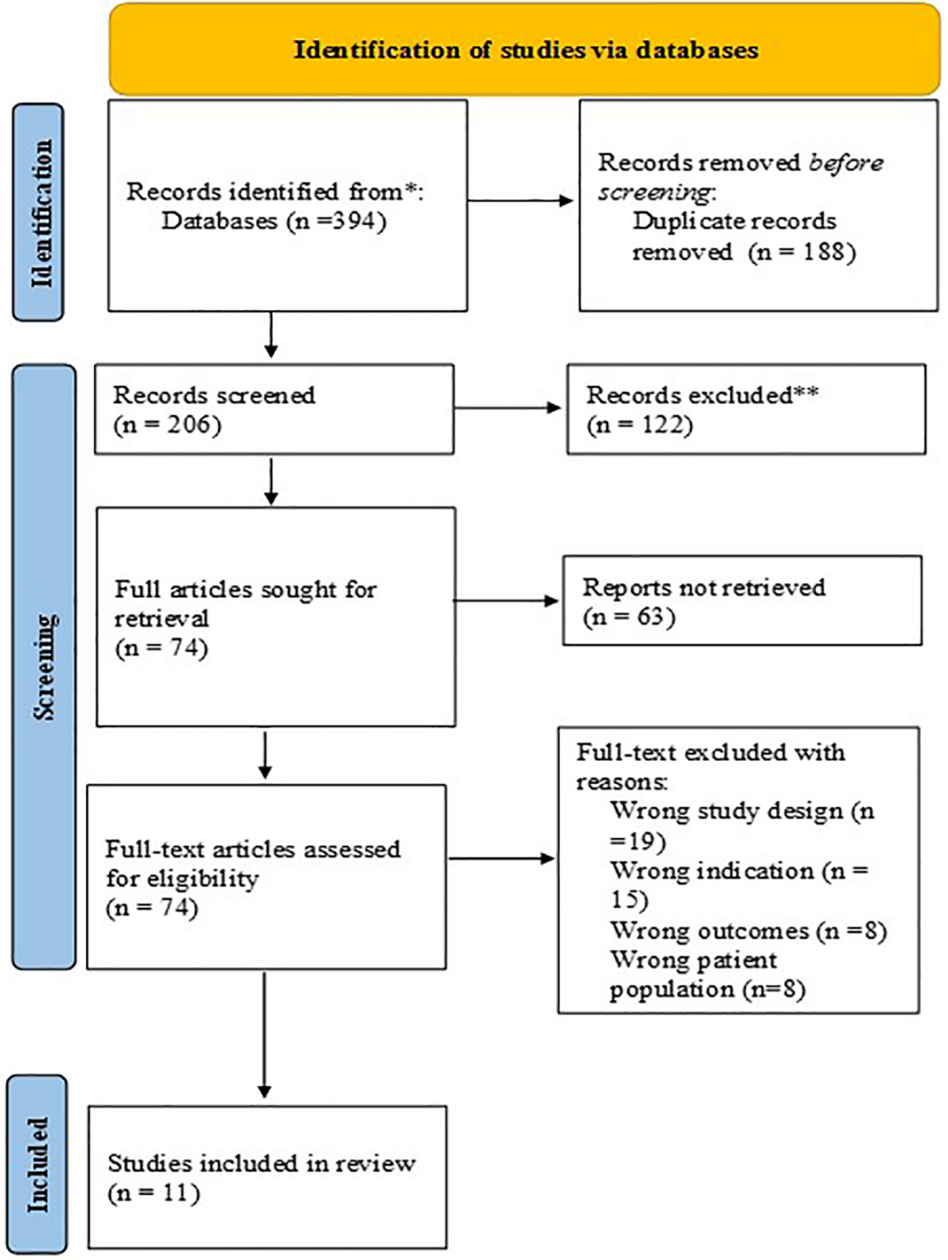
PRISMA flow diagram depicting the flow of information screened and reviewed.

Figure 2 indicates a PRISMA study flow diagram describing how papers were selected for inclusion. Table 3 provides a summary of the 11 studies that were appraised in this review. Table 1 describes gender transformative considerations identified in the 11 studies. The studies are diverse in terms of sample size, sample population, study design and mHealth delivery system. Sample sizes ranged from 18 to 2,550 participants. Participants included pregnant and postpartum women.

**Table 3:** Summary of studies selected for review.

Study authors, title, year	Country	Number of participants	Sample of population	Study design	mHealth delivery system
Ampt et al., (28) Effect of a mobile phone intervention for female sex workers on unintended pregnancy in Kenya	Kenya	786 women	−93 randomly selected sex-work venues in two sub-counties of Mombasa, Kenya (Kisauni and Changamwe) −401 participants from the intervention group from 47 venues −385 participants from the control group from 46 venues	A two-arm, cluster-randomized controlled trial study	SMS text messages
Dev et al., (29) Acceptability, feasibility, and utility of a mobile health family planning decision aid for postpartum women in Kenya	Kenya	25 postpartum women and 17 Family planning providers	Twelve (48%) postpartum women were from rural MCH clinics from the Nyanza region, and 13 (52%) were from urban clinics in Nairobi; 15 (60%) were adolescents and young women between the ages of 14–21.	A cross-sectional qualitative study	An interactive mobile application
Flax et al., (30) Group cell phones are feasible and acceptable for promoting optimal breastfeeding practices in a women's microcredit program in Nigeria.	Nigeria	375 postpartum women participants in total: Quantitative study *n* = 195 Qualitative study *n* = 162		Mixed method: interview and Focus group discussions	SMS text messages and voice message
Harrington et al., (31, 32) Engaging men in a mHealth approach to support postpartum family planning among couples in Kenya: a qualitative study.	Kenya	50 pregnant and postpartum women and men	35 men and 15 women Participants were chosen from two counties in the Nyanza region of Western Kenya. These hospitals serve a primarily low- to middle-income rural population, the majority of whom identify with the Luo ethnic group.	Qualitative study: Focus group discussions	SMS messages
Isler et al., (33) “If he sees it with his own eyes, he will understand”: how gender informed the content and delivery of a maternal nutrition intervention in Burkina Faso.	Burkina Faso	78 pregnant or breastfeeding women and 8 Community Health Workers	The study sampled the catchment areas of two urban and four rural health centres. Healthcare providers identified pregnant and breastfeeding mothers to participate in the study	Qualitative study: focus group discussion	Video was shown on a tablet
Lund et al., (34) Mobile phones as a health communication tool to improve skilled attendance at delivery in Zanzibar: a cluster-randomized controlled trial.	Tanzania	2,550 pregnant women	1,311 women were allocated to the mHealth interventions and 1,239 women were allocated to standard care, i.e. no phone intervention. Participants were recruited from 24 primary healthcare facilities.	Cluster-randomized controlled trial with	SMS
Onono et al., (35) Narratives of women using a 24-h ride-hailing transport system to increase access and utilization of maternal and newborn health services in rural western Kenya: a qualitative study.	Kenya	18 postpartum women	Emphasis was placed on ensuring participants were from different socioeconomic backgrounds. Women were on average 27 years old, married and multiparous with over half having secondary education.	A qualitative study using in-depth interviews (IDIs) as the primary data collection method.	A mobile phone app
Parkes-Ratanshi et al., (36) Low male partner attendance after syphilis screening in pregnant women leads to worse birth outcomes: The Syphilis Treatment of Partners (STOP) randomized control trial	Uganda	290 pregnant women	144 participants enrolled in SMS reminder, 146 enrolled in telephone call reminder	The sample was taken from the IDI clinic located within urban Kampala, where 220 pregnant women are seen at the ANC per month, 5.1% of whom tested positive for syphilis, and the Mulago Hospital ANC, where, on average, 2,000 pregnant women are seen monthly, with 2.4% testing positive for syphilis.	Text messages and telephone calls
Schwartz et al., (37) Acceptability and Feasibility of a Mobile Phone-Based Case Management Intervention to Retain Mothers and Infants from an Option B + Program in Postpartum HIV Care	South Africa	50 pregnant women	HIV-infected, pregnant women attending antenatal care (ANC) at Witkoppen Health and Welfare Centre (WHWC)	Quantitative cohort study	SMS text messages and phone calls
Skinner et al., (38) User assessments and the use of information from MomConnect, a mobile phone text-based information service, by pregnant women and new mothers in South Africa.	South Africa	46 women (pregnant women and new mothers)	Participants were purposively selected from 15 facilities in five provinces—Western Cape, KwaZulu-Natal, Free State, Gauteng and Mpumalanga—which were purposively selected to represent different language and cultural groups in South Africa. Within each province, three facilities were purposively selected among those serving large urban communities and those serving semirural communities or villages.	Qualitative study: interviews and focus groups.	Text-based information service
Trafford et al., (39) Reported infant feeding practices and contextual influences on breastfeeding: qualitative interviews with women registered to MomConnect in three South African provinces	South Africa	115 pregnant and postpartum women	Women over the age of 18 with an expected due date of delivery between April and October 2017 were recruited -using a series of SMS surveys sent out in English and the most common local language in each region—isi-Zulu (KwaZulu-Natal, Gauteng) or Sesotho	Qualitative study: included IDIs and FGDs.	Text messages via standard short message service (SMS) on mobile phones

Study authors, date	Intervention description	Study aims	Intervention outcomes	Major findings	Limitations
Ampt et al., (28)	WHISPER is an SMS intervention. Text messages in English are delivered two to three times per week for 12 months. Message content in the intervention group focused on promoting contraception, particularly long-acting reversible contraception. Message content in the control group focused on promoting nutritional knowledge and practices, including food safety, preparation, and purchasing.	This study aimed to assess the effectiveness of the intervention to reduce the incidence of unintended pregnancy among sex workers in Kenya.	Incidence of unintended pregnancy. Long-acting reversible contraceptive use. Dual contraceptive-method use. Contraceptive knowledge.	The intervention did not have a clinically significant effect on unintended pregnancies despite being developed in consultation with participants (sex workers). The intervention was associated with improved knowledge about contraception, particularly intrauterine devices, as well as increased use of dual-method contraception.	Short-lived measurement of outcomes The study did not account for selective loss of pregnancy among sex workers.
Dev et al., (29)	iMACC is a client-facing mobile application designed to provide systematic, yet personalized, contraceptive counselling to postpartum women. iMACC combines images and text in a heuristic approach to provide tailored contraceptive counselling to women.	This study aimed to evaluate the acceptability and feasibility of the self-administered iMACC decision aid	Behaviour change	Participants indicated that the decision aid was easy to use but challenges arose as to low literacy levels posing a barrier to understanding and using the app. – The app was perceived as a confidential decision aid. – Improved knowledge of contraception with a personalized approach.	The evaluation study did not completely reflect the underlying view of participants towards the FP decision aid.
Flax et al., (30)	Weekly cell phone breastfeeding text and voice messages were delivered to women in groups during group breastfeeding learning sessions	This study aimed to examine the feasibility and acceptability of using group cell phones to deliver cell phone messages within a multi-component breastfeeding promotion intervention. The study also aimed to test the association between participation in small groups and reported breastfeeding practices. Methods	Increased the odds of exclusive breastfeeding. Shift social norms around breastfeeding practices.	Group phones were described as acceptable and functional. Participants reported improved status of the phone holder because she was seen as a leader. Some non-phone recipients wanted their own phones. Health information was passed along to family and friends.	Giving out cell phones to groups is not widely sustainable.
Harrington et al., (31, 32)	The intervention is an interactive SMS program that supports women and couples in contraceptive decision-making, method initiation and continuation.	The study aimed to explore men's and women's perspectives on using SMS to facilitate postpartum family planning counselling in Kenya. It also aimed to engage men in family planning decision-making.	Family planning initiation and continuation	Men strongly desire inclusion in FP decision-making. Men participants indicated that they were open to receiving family-panning related SMS, and discussed multiple advantages of using SMS dialogue for FP counselling. The majority of men and women in FGDs felt that receiving SMS about FP could promote improved communication with their partners.	There may have been selection bias of married women participants. Issues of access to maternal healthcare may be different for non-married women.
Isler et al., (33)	Healthcare providers visited pregnant and breastfeeding mothers at home to show them a set of maternal nutrition videos on a tablet.	To examine how gender affects the content and delivery of a nutrition-focused intervention in Burkina Faso.	Gender-sensitive nutrition intervention	Some mothers said that while they appreciated being shown the videos, they felt powerless to make nutritional changes without the support of their male partners. Healthy promotion in areas such as healthy nutrition, pregnancy and breastfeeding is largely considered a mother's domain. Mothers divulged feeling embarrassed at the prospect of discussing certain topics with men, including intra-household dynamics around nutrition financing.	The study did not interview fathers therefore, it lacks the perspectives of men and their role in maternal and child nutrition.
Lund et al., (34)	An automated short messaging service (SMS) system providing mothers with unidirectional text messaging, and a mobile phone voucher system providing the possibility for direct two-way communication between mothers and their primary healthcare providers	The study aimed to evaluate the use of mobile phones to bridge the communication gap between pregnant women and health providers. The study also aimed to increase skilled delivery attendance in a setting with scarce resources.	Increased skilled delivery attendance amongst pregnant women.	60% of women in the intervention group delivered in the presence of a skilled birth attendant, compared to 47% of women in the control group. However, the intervention did not improve skilled birth attendance in rural areas. Not owning a mobile phone was associated with lower odds of skilled delivery attendance.	Methodological limitation in study design and external validity.
Onono et al., (35)	MAcess is a phone app with the following functions 1) A free SMS-based service that involves sending personalized trimester-based texts to mothers and reminders to use ante- and postnatal care services; 2) An interactive chat service known as m-convo; and 3) 24-h transport navigator system, through which women can request transport	The study aimed to explore how pregnant and postnatal women made decisions regarding care-seeking for pregnancy and childbirth; the processes of getting care from home to the hospital as well as their perceptions on how the MAccess intervention affected their pregnancy and childbirth care-seeking and utilization experience.	Reduction in maternal mortality through frequent communication with healthcare providers and access to reliable transport services.	The bidirectional text messages influenced the decision-making process for the mothers by increasing their knowledge of danger signs, individual birth plans, possible complications, and immediate post-delivery neonatal care. The bidirectional SMS system offered by the MAccess mitigated the challenge of inadequate information. MAccess linked mothers to reliable transport providers to transport them to hospitals. The mHealth innovation was found to be attractive and highly acceptable to women throughout pregnancy and childbirth and helped them navigate the complex and layered individual, infrastructural, and health system factors that put them at risk of adverse maternal and newborn outcomes.	The gatekeeping role of nurses who influenced participant selection might have introduced a bias.
Parkes-Ratanshi et al., (36)	Weekly text message reminders are sent out to women participants to encourage their partners to get tested for syphilis in clinics. This message lasts up to 8 weeks after women's diagnosis.	The aim of this study was to determine the effectiveness of three partner notification strategies, they include: standard of care (SOC) notifications vs. SOC plus text message reminders vs. SOC plus telephone call reminders.	The proportion of partners who presented at the clinic and received syphilis testing or treatment.	Post-enrollment partner attendance was 18%. This was considered low attendance. There were no significant differences between the methods used (SOC notification slip, telephone call or SMS).	The attendance rate recorded in this study is lower than the national average. Insufficient attention to cultural and systemic barriers faced by women to inform their partners of syphilis status and partner getting tested.
Schwartz et al., (37)	This intervention provides support to HIV-infected women on highly active antiretroviral therapy (HAART) during late pregnancy and for six weeks postpartum. Participants received weekly pre-scripted messages from Case managers during pregnancy (36 weeks) and up to 8 weeks postpartum. Case managers made one pre-delivery and two post-delivery telephone calls to participants during follow-up to discuss delivery plans and postpartum care.	The objective of the study was to assess the acceptability, feasibility and potential for scale-up of the mobile phone-based case management intervention.	Retention of mothers in HIV care.	Findings showed that 98% of participants indicated that it was helpful to have a case manager assigned to support them during their pregnancy and postpartum, and all women indicated that they would recommend the case management program to a friend. The pilot intervention provided support to HIV-infected women on HAART during late pregnancy and for six weeks postpartum to be feasible and was highly acceptable. Post-partum contact at 6 weeks was maintained via cellphone with 88% of enrolled women and via telephone or clinic visits amongst 96% of women.	The study was not powered or designed to assess efficacy. The study only collected prospective data and completed questionnaires with women enrolled in the pilot intervention.
Skinner et al., (38)	MomConnect is a text service provided to pregnant women and new mothers. Women received text messages covering broad areas of child care and health.	The study aimed to describe the experiences of pregnant women and new mothers with MomConnect and assess participants’ perceived value of MomConnect, and obtain suggestions for its further improvement.	Empowered mothers who have skills to care for their children.	The pregnant women and mothers uniformly were highly appreciative of MomConnect. First-time mothers felt that they had a lot to learn and drew support and confidence from the messages. MomConnect met the requirement of being responsive to the target group's needs.	
Trafford et al., (39)	Mom connect was introduced as a strategy to increase breastfeeding rates. Women registered with MomConnect receive health information through text messages and have access to a helpdesk which allows them to ask questions about health services received.	To provide evidence towards a richer understanding of women's practices around breastfeeding, key influences on their decision-making, and what might facilitate or prevent the enactment of their breastfeeding intentions.	Increased breastfeeding initiation	MomConnect messages (often used in conjunction with health workers’ advice) were noted as a very valuable source of information that supported women's knowledge and decision-making. Women found MomConnect empowering and used the messages to teach their peers.	Many of the women were unemployed. Evidence shows an association between low rates of employment and high rates of breastfeeding.

#### Key finding 1: SMS-based services are the most common mHealth delivery system

Study designs included six qualitative studies (29, 31, 33, 35, 38, 39); three randomized control trials (28, 34, 36); and two mixed method studies (30, 37). The most common mHealth delivery system was text messages via standard short message service (SMS) on mobile phones (28, 30, 31, 33, 34, 36–39), the other approaches used interactive mobile apps (29, 35). Outcomes of interventions to improve maternal health varied across the studies. Three studies focused on reducing unintended pregnancies through the promotion of contraceptive use (28, 29, 31). Two studies focused on improving breastfeeding among postpartum mothers (30, 39), two studies aimed to increase women's access to skilled health personnel during pregnancy, childbirth and postpartum (34, 35). One study targeted improved and adequate nutrition among pregnant and breastfeeding mothers (33). Two studies aimed to prevent and manage sexually transmitted diseases among pregnant and postpartum women (36, 37).

#### Key finding 2: few studies substantively incorporated gender transformative dimensions in their study aims

Findings responding to the first research question indicate that all studies included at least one of the six gender transformative considerations but only three studies substantively incorporated gender transformative dimensions in the aim of their study (31, 33, 36). One study aimed to examine how gender impacts the content and delivery of a nutrition intervention focused on mothers (33). Another study aimed to involve men and women in discussions around family planning education and decision-making (31). Finally, one aimed to encourage men (partners of pregnant women) to get tested and treated for sexually transmitted infections (STI) to decrease incidences of STIs in women during pregnancy (36). For the rest of the studies, gender considerations were not explicitly stated but treated tangentially within the mHealth program's design, implementation, or evaluation.

#### Key finding 3: a common gender consideration was of women's specific needs

Two studies each included 3 gender transformative considerations (31, 33), four studies included 2 gender transformative considerations (28–30, 38), the rest of the studies only had one (34–37, 39). Two studies included strategies to promote gender equality (29, 30). Strategies to promote gender equality included closing the knowledge gaps about family planning between men and women and improving women's financial stability through microcredit programs. Most of the gender considerations fell under the category of considering women's specific needs.

One study indicated consideration for women's specific needs by co-designing a mHealth program aimed at improving contraceptive knowledge and use with the target population, this approach enabled the researchers to integrate the needs of the women into their program (28). Four studies conducted preliminary research with their target population and designed mHealth programs based on identified needs; One study identified knowledge gaps as a barrier to women's decision-making about family planning and subsequently designed a mHealth program to educate women on contraceptive choices and enhance their decision-making regarding family planning (29). Another study was informed by formative studies that linked limited decision-making support to the use of pregnancy care. Consequently, the researchers designed a mHealth intervention to support women's decision-making (35). Two studies considered women's specific needs by first conducting a formative study that revealed a gender gap in women's access to mobile devices (30, 34). The researchers designed their mHealth program to optimize women's participation even without individual mobile phone ownership or with low literacy status. Women were able to participate either using group cell phones or shared cell phones. In addition, one study considered women's specific needs by being mindful of their schedules, domestic responsibilities, and transportation challenges during their study (33). The mHealth intervention, which involved presenting nutrition information through an interactive video, was delivered to women at their doorstep. One study met the needs of pregnant and postpartum women with HIV through the provision of HIV counselling and support (37). This study also guaranteed women's privacy by protecting their sensitive health information. Another study delivered mHealth programs at no cost to low-income pregnant and new mothers (38).

#### Key finding 4: men have a pivotal role in maternal health

Turning to another category of gender transformative consideration, the aims or outcomes of three studies addressed causes of gender-based health inequities. One study indicated that SMS messages from the mHealth program challenged social norms around the use of contraceptives and pregnancy risk (31). Another mHealth program sought to enhance adequate nutrition among pregnant women by involving men who are often major decision-makers in maternal nutrition (33). Finally, one study identified untreated men partners as primary drivers of syphilis in women during pregnancy, therefore the program targeted pregnant women's partners to test for and treat syphilis symptoms (36). Three studies included considerations under the category of seeking to transform harmful gender norms. One acknowledged that women faced an increased likelihood of gender-based violence due to participating in the mHealth study (28). The authors arranged for the protection of women by providing urgent medical care where necessary and garnered support for and protection of women from community mobilizers. In another study, transforming gender norms also meant educating couples (men and women) about family planning through SMS text messages and supporting their joint decision-making (31). One study encouraged women to share text messages on pregnancy and child care with their spouses (38). Within these messages, the dangers of domestic violence were emphasized. Women reported a sense of support from receiving and sharing messages with their spouses.

Gender considerations in two studies indicated strategies to foster progressive changes in power relationships between women and men; One study engaged men in family planning education and decision-making support and also employed innovative strategies to go beyond couples or individual interventions but also community-level engagement to improve knowledge on family planning (31). Another study fostered progressive changes in power relationships between women and men by legitimizing the importance of breastfeeding through SMS text messages (39). Men's disapproval of breastfeeding deterred women from breastfeeding, however, women indicated that receiving and sharing the text messages from the mHealth program enabled them to resist pressure from men and encouraged breastfeeding.

#### Key finding 5: findings from gender transformative mHealth programs indicate positive results overall

Studies included in this review showed positive results overall. One mHealth program aimed at altering postpartum women's habits and behaviour toward contraceptive use saw improvements in women's knowledge of contraceptives (29). Another mHealth program aimed at increasing exclusive breastfeeding practice among postpartum women was described as acceptable and functional by the participants (30). Including men in a mHealth family planning program for postpartum women improved their communication with their women partners around contraceptive use (31). One study saw an increased rate of skilled delivery attendance amongst women participants as a result of the mHealth program (34). A mHealth program that provided a 24-hour transport navigator system reported improved maternal access to skilled pregnancy care including virtual communications with their healthcare providers (35). In another mHealth study, an intervention that aimed to retain and support HIV-infected mothers was perceived as helpful and supportive by participants (37). In two studies, participants demonstrated the positive impact of MomConnect, a mHealth program for pregnant and postpartum women. The mHealth program was responsive to the needs of new mothers and served as an empowering force toward positive breastfeeding practices for women (38, 39).

However, not all studies reported positive findings. One mHealth program was developed jointly with target participants in order to reduce incidents of unintended pregnancies, however, the program showed no clinically significant effect on unintended pregnancies among participants (28). Additionally, other studies indicated the need for a more explicit focus on gender consideration in a mHealth program's design or implementation. One study targeted pregnant and breastfeeding women to educate them on adequate nutrition during pregnancy (33). While participants improved their knowledge of appropriate nutrition during pregnancy and postpartum, they were powerless to make any nutritional changes without support from their male partners. Similarly, another study aimed at encouraging the testing and treatment of STIs among male partners of pregnant women indicated a limited or low effect of the program (36). The authors pointed to insufficient gender considerations in the mHealth design and implementation. In another mHealth study, limited considerations of intersecting domains of disadvantages, specifically gender and geographic location, led to the exclusion of the most vulnerable of women (34). In the study which aimed to improve women's access to skilled birth attendants, women were able to participate in the mHealth program regardless of phone ownership or literacy status. The study saw improvements in access to skilled birth attendants in urban areas but failed to reach rural women who were in dire need of skilled attendants during childbirth (34).

#### Key findings 6: gender considerations and maternal health outcomes

Furthermore, this review offered some evidence on how gender considerations influenced maternal health outcomes. In considering gender differences, one study identified crucial knowledge gaps that hampered post-partum women's use of modern contraceptives (29). Women's unmet need for contraception was exacerbated by their limited knowledge on contraceptives. Through the mHealth program, women showed improved knowledge and more thorough understanding of contraceptives. The authors highlighted the potential of increased knowledge to improve contraceptive use among postpartum women (29). In another study, specific considerations for women's limited phone ownership increased their odds of exclusive breastfeeding for up to 6 months (30). Through the use of group cell phones, women received text messages that promoted optimal breastfeeding practices and were more likely to breastfeed exclusively for the first 6 months. In a similar study aiming to improve breastfeeding rates, text messages shared with women and their families targeted unfavourable social norms (39). Women felt empowered to make breastfeeding choices and to resist pressure against breastfeeding that was often brought about by patriarchal norms. Women in the study reported high rates of breastfeeding. In a study aimed at improving postpartum retention in HIV care, a mobile health program delivered health information and reminder text messages to women directly from their healthcare providers (37). Gender considerations ensured that women's HIV status was not disclosed in those text messages. This study showed improved communication between women and healthcare providers, especially among women who wanted to maintain the privacy of their health information. Overall, interactions with healthcare providers contributed to women's retention in HIV care (37).

## Discussion

To our knowledge, this is the first systematic review reporting evidence on gender transformative approaches in mHealth programs for maternal healthcare in sub-Saharan Africa. The study highlights the various approaches to integrating gender transformative approaches in mHealth studies in line with the WHO's definition of a gender transformative approach to health programs (18). The findings indicate that while most of the evidence of transformative approaches centred on considering women's specific needs, there was a limited focus on advancing gender equality. No study covered the entire categories of gender transformative approaches and a few studies included approaches from a maximum of three categories.

Our findings with the most significant policy concern are the limited number of mHealth programs with an explicit focus on gender transformative considerations. Gender transformation was not necessarily central to most mHealth programs although they manifested during the study. This highlights the need for an explicit and intentional focus on gender considerations and the promotion of gender equality in mHealth programs for maternal healthcare. Our findings indicated that consideration of only one gender target is often insufficient to effect change. For instance, in a mHealth study to improve nutrition during pregnancy and early childhood, women who were target participants improved their knowledge of adequate nutrition, but improved awareness did not translate into appropriate action because men were not actively engaged in the program. Improving nutrition by targeting women alone presupposes their access to financial resources and decision-making power. In line with this insight are findings from studies in sub-Saharan Africa that illuminate the gender power dynamics inherent in the context of women's nutrition and health (48). The study highlighted the importance of considering women's broader social, cultural, and economic realities and involving men in health interventions.

Engaging men in and of itself is not a panacea as illustrated by another mHealth study in our review. The study observed that men were the predominant drivers of syphilis in pregnancy and encouraged women to recruit their men partners to test for and treat STIs (36). The study saw poor attendance from men and contended that gender-based barriers prevented effective communication between partners. A similar study in Congo highlighted the dangers of poorly designed mHealth programs for engaging men in maternal healthcare (49). The study, jointly targeting men and women, was designed to bridge the knowledge gap around modern contraceptives but instead saw higher participation among men than women. The study failed to account for the digital gap whereby men were often primary users of technology. As evidenced by our findings and the broader literature, engaging men in maternal health requires a deeper consideration of men's privilege and power over women (4). Men need to be engaged meaningfully in maternal health programs.

Encouragingly, most of the studies showed positive findings in advancing women's access to maternal healthcare services. Specifically, our findings show evidence of positive outcomes in multi-sectorial approaches to enhancing maternal health. One study from our review integrated breastfeeding promotion into a microcredit program for pregnant mothers in Nigeria. The aim was to improve women's financial stability while supporting breastfeeding through a mHealth program. Similar studies in the literature demonstrate how multi-pronged gender transformative programs for maternal health led to positive health outcomes. A mHealth program in Kenya empowered women in informal employment sectors to save for maternal health expenditure as well as improve their knowledge of maternal healthcare (50, 51). When financially empowered, women are more likely to seek and adhere to skilled maternal health care (50, 51). Similarly, programs to redress anemia in pregnant women in Burkina Faso and DRC went beyond nutrition-related activities to involve women in sanitation supply chain initiatives, enhance women's leadership in communities and shed light on gender-based violence (52). These examples show a recognition of the complex and interconnected factors that determine maternal health. They also highlight the potential of mHealth to facilitate a multisectoral approach to redress maternal mortality and morbidity.

Findings from our study illustrate the influences of gender considerations on maternal health outcomes. Our studies highlight the importance of gender considerations such as acknowledging that women are more likely than men to be digitally excluded. According to the Mobile Gender Gap Report 2021 published by the GSMA, the gender gap in mobile phone ownership in sub-Saharan Africa is at 13%, this translates to 74 million women who do not own a mobile phone (13). Studies have shown that enhancing women's access to mobile devices enhances their participation in mHealth studies, increases their use of maternal healthcare services, and consequently improves maternal health outcomes (24, 53). Our findings show that women's perception of the security of their health information impacts their use of mHealth programs. A mHealth study that guaranteed women's privacy saw increased engagement with the program and subsequent improvement in maternal health outcomes (37). Similar to our findings, evidence from Tanzania shows that positive perceptions of personal privacy and security of a mHealth program enhances pregnant women's participation in the program (54). The study also showed enhanced relationships between women and their healthcare providers.

### Policy implications

Implications of our findings for policy have been interspersed in the discussion. We draw further attention to privacy as a growing concern in digital health especially as it pertains to sensitive health information (55). Disclosing private health information puts women at increased risk of violence (37). Our findings showed strategies for circumventing privacy issues such as purposefully designed mHealth programs that deliver general messages on HIV without divulging women's HIV status. An additional strategy could be the integration of password-protected messages to ensure that only the intended recipient reads messages. Beyond program-level strategies, the Global Strategy on Digital Health advocates for country-level regulatory frameworks to enhance the protection and confidentiality of health data with the use of digital health (56). To address the challenges identified in our findings, gender considerations must be integrated into the planning and implementation of these frameworks. Our findings also indicate the need for improved digital access for women. Addressing issues related to affordability and literacy is key to enhancing women's access to and use of mobile health technologies (57). This will require cross-sectoral collaborations and an explicit focus on gender perspectives in policies and plans for digital health. For instance, subsidizing phones for women and girls and digital literacy programs can overcome gendered access barriers to mobile technologies (57).

An understanding of the existing gender ecosystem maximizes the potential of digital health innovations and minimizes risks particularly as it relates to engaging men in maternal health (58). Our findings show the need for men to be engaged meaningfully in maternal health programs. Strategies to enhance male engagement in sub-Saharan African countries have included the development of male engagement guidelines as evidenced in Tanzania (59). It is important to note that while well-intended, unintended consequences of these guidelines have been shown to present challenges for women. For instance, partner absence during antenatal care visits has resulted in delays in women seeking healthcare or refusal of care by healthcare workers (59). It is important to understand the existing gender ecosystem and assess the unintended consequences of strategies to engage men in maternal health.

The lack of programs that address all the gender-transformative categories indicates the need for a gender objective in each digital health program. Canada's International Development Research Centre (IDRC) recommends that digital health programs in underserved communities should include at least one research question or objective that aims to understand gender issues (58). This will address the noticeable risks of inadequate gender considerations as observed in some of the studies. In our findings, studies with explicit gender objectives also allowed gender to inform further actions in the research process such as data collection. Therefore, beyond having a clear objective to consider gender issues, it is important to maintain a commitment to adapting programs as gender issues become apparent during the course of a program.

### Future research

As demonstrated in our review, there is limited research on gender transformative approaches in mHealth for maternal health in sub-Saharan Africa. Given the transformational potential of digital health, there is a need for more research on how digital health can reduce inequalities for end users, especially women and girls. Research is needed to investigate how gender inequalities shape assumptions, design and implementation of digital health tools. Studies also indicate the need to meaningfully engage gatekeepers in society who enforce gender power relations to enhance the success of digital health programs. Critical gaps identified in our study point to the need for methodologically strong gender transformative studies. There needs to be greater consistency in quality terminology and criteria that accommodate different study contexts. Future studies can investigate the adverse effects of enacting gender transformative approaches including familial tension because of changes in gender dynamics in relationships.

### Strengths and limitations

This study reviewed evidence from both quantitative and qualitative studies thereby uncovering gender as presented from different perspectives. This approach allowed the authors to examine a robust pool of data while gaining insights into users' experiences of gender-transformative mHealth programming. These may not have been possible with only a quantitative or qualitative review of evidence. Despite the generally successful outcomes of gender transformative studies, these studies should be interpreted with caution in light of a few low-quality studies. Low methodological quality scores of studies are indicative of poorly designed studies, therefore, while they may include the relevant gender transformative dimensions, methodological gaps and low-quality studies may exaggerate result outcomes and lead to incorrect inferences. There is a need for more rigorous study designs, especially for mixed methods mHealth studies for maternal healthcare. Furthermore, our analyses of findings indicate strong individual and community-level approaches to gender integration in mHealth programs. Similar approaches have been shown to transform gender norms and health-related outcomes in sub-Saharan Africa (60). However, previous research emphasizes gender transformative approaches at the structural level including legal or policy approaches (60, 61). These approaches have been shown to transform health challenges brought about by gender inequality and achieve effective and sustainable change.

Due to the language limitations of the authors, there was no non-English mHealth study included in this review. The authors may have missed other relevant studies that provide evidence on gender transformative approaches. Another limitation is that while the authors extracted the relevant data using the WHO definition as a guide, the gender transformative parameters were not explicitly stated in the studies. This calls attention to the need for clear reporting guidelines for gender considerations, especially in mHealth research. The literature shows a growing recognition of the importance of consistent standards for reporting gender considerations in health research, however, the deficiencies in the quality of reporting remain an issue (62, 63).

## Conclusion

Digital health has been incorporated across the pregnancy care continuum in efforts to address social determinants of health, improve the quality of care and ensure positive maternal health outcomes. To achieve meaningful impact, gender and digital inclusion must remain a priority in developing, implementing and evaluating digital health programs. This study reviews gender transformative approaches to gender integration in mHealth for maternal health in sub-Saharan Africa. This review adopts the definition offered by the WHO and interprets a gender-transformative digital health program as one that “addresses the causes of gender-based health inequities through approaches that challenge and redress harmful and unequal gender norms, roles, and power relations that privilege men over women”. Considering gender in and of itself is not sufficient because some pathways to gender consideration in health can exploit or accommodate harmful gender norms rather than transform them. While this review affirms that gender transformative approaches in digital health programs are advancing maternal healthcare outcomes, we noted that most programs were not substantively incorporating these considerations into their design, implementation, or evaluation. Implications of our study findings indicate the need for mHealth studies to explicitly acknowledge how power dynamics, values and norms impact maternal health and address these factors throughout the course of a mHealth program.

## Data Availability

The original contributions presented in the study are included in the article/**Supplementary Material**, further inquiries can be directed to the corresponding author.
