# Supplementary material for: Gender transformative approaches in mHealth for maternal healthcare in sub-Saharan Africa: a systematic review
Source: Front Digit Health. 2023 Nov 7;5:1263488. doi: 10.3389/fdgth.2023.1263488 (PMC10662097; doi:10.3389/fdgth.2023.1263488)
Supplement: Supplementary file 2 [file Table2.docx]

**Supplementary file 2: Search Strategy**

Telemedicine OR telehealth OR mHealth OR mobile Health OR electronic* Health OR eHealth OR cell phone* OR cellular phone* OR mobile phone* OR texting OR message* OR SMS OR audio message* OR Interactive Voice OR digital health

**AND**

gender OR sex OR women OR female OR relations OR interaction* OR equity OR inequity OR equality OR inequality OR men OR male OR empower* OR gender role* OR autonomy OR violence OR safety OR literacy OR economic OR mobility OR status OR access OR capacity OR communication* OR gender integration

**AND**

Maternal health OR Pregnan* OR Matern* Matern* services OR birth* OR delivery OR prenatal OR prepartum OR postnatal OR postpartum OR breastfeeding OR obstetric. (title search)

**AND**

sub-Sahara* Africa OR Africa OR West Africa OR East Africa OR Southern Africa

| **Supplementary file 3: Inclusion and exclusion criteria** | | |
| --- | --- | --- |
|  | **Inclusion Criteria** | **Exclusion Criteria** |
| **Participants** | - Women and girls in sub-Saharan countries. - Women and girls who used digital health services for pregnancy, childbirth and postpartum care. - Partners of women or girls who used digital health services for maternal healthcare. | - Health workers who used digital health services for work. - Digital health services for children only. |
| **Context** | - The use of digital health programs to improve maternal health. - Studies that implicitly or explicitly reported observations relating to gender or gender outcomes | - Usage of digital health services by other populations (not women themselves). - Digital health used for data collection not intervention purposes. - Discussion of digital health for the purpose of theory building or critique |
| **Comparison / Control Group** | - No comparison group for this study. |  |
| **Outcome of Interest** | - To present gender transformative dimensions or considerations included in mHealth interventions for maternal healthcare in sub-Saharan African countries. |  |
| **Setting** | - The 42 countries and Island nations in the sub-Saharan African region. They include:   Democratic Republic of Congo, Republic of Congo, Central African Republic, Rwanda, Burundi, Sudan, Kenya, Tanzania, Uganda, Djibouti, Eritrea, Ethiopia, Somalia (including Somaliland), Angola, Botswana, Lesotho, Malawi, Mozambique, Namibia, South Africa, Swaziland  Zambia, Zimbabwe, Benin, Burkina Faso, Cameroon, Chad, Côte d'Ivoire, Equatorial Guinea, Gabon, The Gambia, Ghana, Guinea, Guinea-Bissau, Liberia, Mali, Mauritania, Niger, Nigeria, Senegal, Sierra Leone, Togo, Cape Verde, Comoros, Madagascar, Mauritius, São Tomé and Príncipe, Seychelles | - Countries not included in the sub-Saharan African region. |
| **Study Design** | - Qualitative, Quantitative, and mixed methods studies. - Peer reviewed full-text research papers, published in English. | - Reviews, editorials and commentaries, discussions, theses or dissertations and other gray literature. - Articles published in any other languages other than English. |

| **Supplementary file 4: List of excluded studies** | | |
| --- | --- | --- |
|  | **Author/Year/Country** | **Reasons for exclusion** |
|  | Abajobir et al., 2021, | Wrong study design |
|  | Adam et al., 2019, South Africa | Wrong patient population |
|  | Adam et al., 2019, South Africa | Wrong patient population |
|  | Afutendem et al., 2019,Cameroon | Wrong study design |
|  | Amoah et al., 2016, Ghana | Wrong indication |
|  | Andreatta et al., 2011, Ghana | Wrong patient population |
|  | Clouse et al., 2020, South Africa | Wrong study design |
|  | Clouse et al., 2015, South Africa | Wrong study design |
|  | Colaco et al., 2017, Guinea | Wrong outcomes |
|  | Cole-Ceesay et al., 2010, Gambia | Wrong intervention |
|  | Coleman et al., 2017, South Africa | Wrong indication |
|  | Coleman et al., 2020, South Africa | Wrong outcomes |
|  | Constant et al., 2016, South Africa | Wrong indication |
|  | Daskilewicz et al., 2015, South Africa | Wrong intervention |
|  | Dryden-Peterson et al., 2015, Botswana | Wrong indication |
|  | Frellsen et al., 2016, Ethiopia, Ghana, and Sierra Leone | Wrong study design |
|  | Geldsetzer et al., 2016 | Wrong study design |
|  | Gilles et al., 2011, Malawi | Wrong study design |
|  | Godefay et al., 2016, Ethiopia | Wrong indication |
|  | Gomba et al., 2019, South Africa | Wrong study design |
|  | Gomperts et al., 2012, | Wrong indication |
|  | Hackett et al., 2018, Tanzania | Wrong indication |
|  | Haddad et al., 2015, Malawi | Wrong intervention |
|  | Jennings et al., 2015, Nigeria | Wrong study design |
|  | Job et al., 2021, South Africa | Wrong patient population |
|  | Kabongo et al., 2019, South Africa | Wrong study design |
|  | Kayongo et al., 2019, Uganda | Wrong intervention |
|  | Kilonzo et al., 2017, Kenya | Wrong study design |
|  | Kola et al., 2021, Nigeria | Wrong intervention |
|  | Laing et al., 2020, Gambia | Wrong outcomes |
|  | Lamont et al., 2016 | Review article |
|  | Lori et al., 2012, Liberia | Wrong patient population |
|  | Mbaruku et al., 2018, Tanzania | Wrong outcomes |
|  | Mercader et al., 2017, Uganda | Wrong outcomes |
|  | Murphy et al., 2014 | Review article |
|  | Mwase et al., 2020, Uganda | Wrong indication |
|  | Nachega et al., 2016, South Africa | Wrong study design |
|  | Ndlovu et al., 2019, South Africa | Wrong indication |
|  | Obasola et al., 2015 | Review article |
|  | Odeny et al., 2014, Kenya | Wrong indication |
|  | Odetola et al., 2018, Nigeria | Wrong study design |
|  | Odetola et al., 2016, Nigeria | Wrong indication |
|  | Omonaiye et al., 2020 | Review article |
|  | Onono et al., 2021, Kenya | Wrong outcomes |
|  | Owili et al, 2018, 23 African countries | Wrong intervention |
|  | Parkes-Ratanshi et al., 2018, Uganda | Duplicate study |
|  | Petow et al., 2012 | Wrong indication |
|  | Phillips et al., 2019, South Africa | Wrong study design |
|  | Pintye et al., 2020, Kenya | Wrong indication |
|  | Purcell-Jones et al., 2019, South Africa | Wrong indication |
|  | Ramachandran, 2013, | Wrong patient population |
|  | Ronen et al., 2018, Kenya | Wrong outcomes |
|  | Rotheram-Borus et al., 2011, South Africa | Wrong study design |
|  | Sheth et al., 2010, Malawi | Wrong outcomes |
|  | Tull et al., 2019, Kenya, Uganda | Wrong study design |
|  | Ukachi et al., 2019, Nigeria | Wrong study design |
|  | Unger et al., 2018, Kenya | Wrong indication |
|  | vanHeerden et al., 2010, South Africa | Wrong patient population |
|  | vanHeerden et al., 2013, South Africa | Wrong study design |
|  | Vilaplana et al., 2020, Madagascar | Wrong study design |
|  | Vrazo et al., 2018 | Review article |
|  | Willcox et al., 2019, Ghana | Wrong patient population |
|  | Zunza et al., 2017, South Africa | Wrong study design |
